# Thoracoabdominal CT Angiography Detection of Anterior Mitral Leaflet Perforation in Recurrent Infective Endocarditis After Serial Echocardiograms: A Case Report

**DOI:** 10.7759/cureus.115618

**Published:** 2026-09-01

**Authors:** Ersin Ozturk, Cagri Yurtsever, Muhammad Awiwi, Cihan Duran

**Affiliations:** 1 Radiology, Kadikoy Medicana Hospital, Istanbul, TUR; 2 Radiology, Lehigh Valley Hospital, Behtlehem, USA; 3 Radiology, Rutgers New Jersey Medical School, New Jersey, USA; 4 Radiology, The University of Texas Health Science Center at Houston, Houston, USA

**Keywords:** aortic graft infection, computed tomography angiography, echocardiography, infective endocarditis, leaflet perforation, mitral valve, prosthetic valve endocarditis, vegetation

## Abstract

Infective endocarditis (IE) can cause destructive valvular complications, including leaflet perforation and vegetation. Echocardiography remains the primary imaging modality for valvular assessment; however, computed tomography angiography (CTA) may reveal clinically important cardiac findings when performed for other urgent indications. We report the case of a 70-year-old male with recurrent IE, prior prosthetic aortic valve endocarditis first diagnosed in 2022, multiple aortic valve and aortic root operations, thoracic endovascular aortic repair with a left subclavian stent, prior aortic graft infection, methicillin-resistant *Staphylococcus aureus* (MRSA) endocarditis and bacteremia, chronic thoracic osteomyelitis, chronic bilateral hip prosthetic joint infection, heart failure with reduced ejection fraction, severe mitral regurgitation, complete heart block with a permanent pacemaker, paroxysmal atrial fibrillation, and multiple additional comorbidities. He was transferred for hemoptysis and fever, and thoracoabdominal CTA was obtained to evaluate for an aorto-bronchial fistula. The CTA showed no evidence of aorto-bronchial fistula and demonstrated decreased peri-graft fluid compared with prior imaging. Systematic cardiac review demonstrated a focal anterior mitral leaflet defect with an adjacent 0.9 cm hypoattenuating lesion along the left atrial surface, suspicious for leaflet perforation with vegetation. The abnormality had not been reported on two transesophageal echocardiographs (TEEs) and four transthoracic echocardiographs (TTEs) performed earlier in the same year. This case highlights the complementary value of careful cardiac valve assessment on non-coronary CTA in patients with recurrent endocarditis and complex prosthetic cardiovascular material.

## Introduction

Infective endocarditis (IE) is a high-morbidity infection of the endocardial surface that may involve native valves, prosthetic valves, vascular grafts, and intracardiac devices. Patients with prior valve replacement, prior IE, vascular graft infection, chronic osteomyelitis, and indwelling cardiac devices represent a particularly complex diagnostic population because infection may recur, involve multiple cardiovascular structures, or produce subtle destructive complications. Current diagnostic frameworks emphasize multimodality imaging, with echocardiography remaining central and cardiac CT increasingly recognized as a complementary modality [1,2].

Valvular destruction in IE includes vegetation, leaflet perforation, and other valvular or perivalvular complications. Leaflet perforation is clinically important because it may substantially worsen regurgitation, alter surgical risk, and indicate active or previously destructive infection. Color Doppler echocardiography may demonstrate a regurgitant jet traversing the leaflet defect, helping confirm the presence and hemodynamic significance of perforation. Transesophageal echocardiography (TEE) is usually more sensitive than CT for small vegetations and leaflet perforations, but dedicated ECG-gated cardiac CT can provide important anatomic information, especially when prosthetic material, aortic graft complications, or extracardiac manifestations are being evaluated [3-5].

We describe a patient with recurrent IE and a complex aortic surgical history in whom a routine nongated thoracoabdominal CTA, performed for hemoptysis and suspected aortobronchial fistula, showed no fistula but incidentally revealed anterior mitral leaflet perforation with an adjacent suspected vegetation. The case underscores the importance of systematically reviewing cardiac valves on nongated, non-cardiac CTA performed for other clinical indications in high-risk endocarditis patients.

## Case presentation

Clinical history

A 70-year-old male was transferred on July 31, 2026, for evaluation of hemoptysis and fever. His cardiovascular history was highly complex. He had undergone bioprosthetic aortic valve replacements in 2008, 2016, and 2021; an aortic root replacement in 2016; a redo aortic root replacement in July 2022; and zone 2 thoracic endovascular aortic repair (TEVAR) with a left subclavian artery stent in October 2022. His infectious history included recurrent endocarditis, with the first diagnosis of prosthetic aortic valve endocarditis made in 2022, after which he underwent operative management. The pathology report from the 2022 operation was not available for review. Additional clinical history included prior infection of TEVAR or infected aortic graft, prior methicillin-resistant *Staphylococcus aureus* (MRSA) endocarditis and bacteremia, chronic thoracic osteomyelitis, chronic bilateral hip prosthetic joint infection on suppressive antibiotics, heart failure with reduced ejection fraction, severe mitral regurgitation, complete heart block treated with a permanent pacemaker, paroxysmal atrial fibrillation, asthma, hypertension, prior cerebrovascular accident, bladder cancer, and diabetes mellitus.

Prior echocardiographic evaluation

A key feature of this case was the discrepancy between the serial echocardiographic reports and the index volumetric CTA obtained during the current presentation. Before the current presentation, the patient reportedly underwent two TEE and four transthoracic echocardiographic (TTE) examinations. None of the available echocardiographic reports described anterior mitral leaflet perforation. This was interpreted as an unreported or previously unrecognized abnormality rather than evidence that the abnormality was definitely absent on those examinations, because the original cine loops and Doppler images were unavailable for direct retrospective review.

CTA technique and vascular findings

Volumetric contrast-enhanced CTA of the chest, abdomen, and pelvis was obtained with axial, coronal, sagittal, and maximum-intensity-projection reconstructions. The clinical indication was evaluation of hemoptysis and suspected aorto-bronchial fistula in the setting of complex aortic graft history and prior graft infection. The index volumetric CTA was compared with CT examinations performed one day and approximately four months before the current presentation.

Postoperative changes of aortic valve replacement, ascending aortic repair, and TEVAR extending from the proximal aortic arch to the proximal descending thoracic aorta were present. A left subclavian artery stent extended into the aortic arch stent graft. Fluid surrounding the ascending aortic graft and proximal aortic arch at the level of the common trunk of the brachiocephalic artery and left common carotid artery anastomosis was again seen but had decreased compared with the CTA examination conducted four months prior to the current presentation, measuring up to 6.1 cm compared with 7.4 cm previously. No pseudoaneurysm or extraluminal contrast was identified adjacent to the brachiocephalic/left common carotid artery anastomotic site, and there were no CTA findings to suggest aorto-bronchial fistula.

Cardiac, airway, and additional findings

Systematic review of the cardiac structures demonstrated cardiomegaly with biventricular and left atrial dilation. A focal defect was identified in the anterior mitral leaflet, consistent with leaflet perforation (Figure 1). Adjacent to the left atrial surface of the anterior mitral leaflet, there was an approximately 0.9 cm hypoattenuating filling defect, suspicious for vegetation in the clinical context of recurrent endocarditis (Figure 1). Mild mitral annular calcification and scattered coronary arterial calcifications were present (Figure 2). Decreased attenuation of the blood pool suggested anemia.

**Figure 1:**
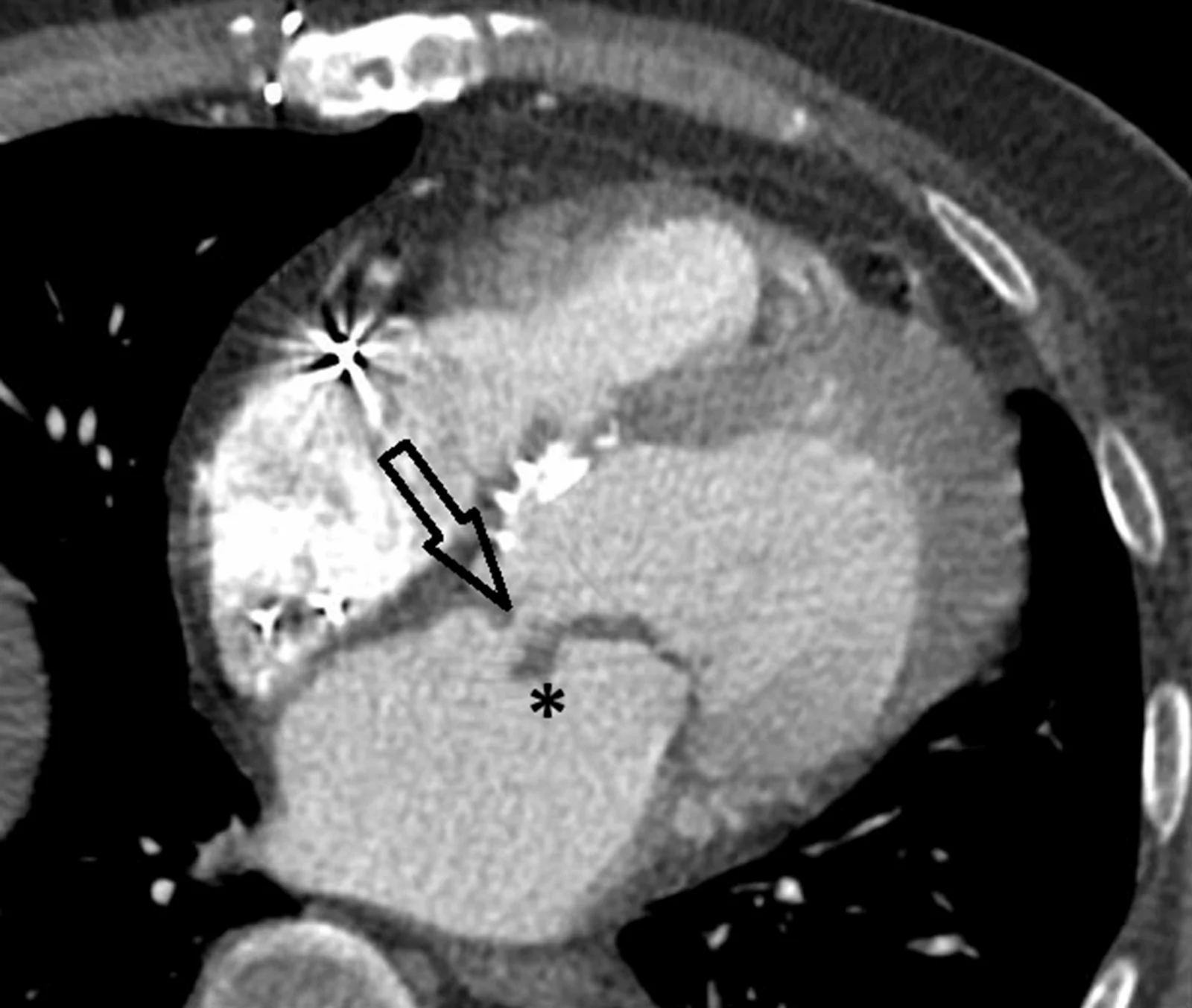
Contrast-enhanced axial CTA image centered on the mitral valve The arrow indicates a focal discontinuity/defect of the anterior mitral leaflet, with an adjacent hypoattenuating lesion (asterisk) along the left atrial surface, suspicious for leaflet perforation with vegetation in the setting of recurrent infective endocarditis. CTA: Computed tomography angiography

**Figure 2:**
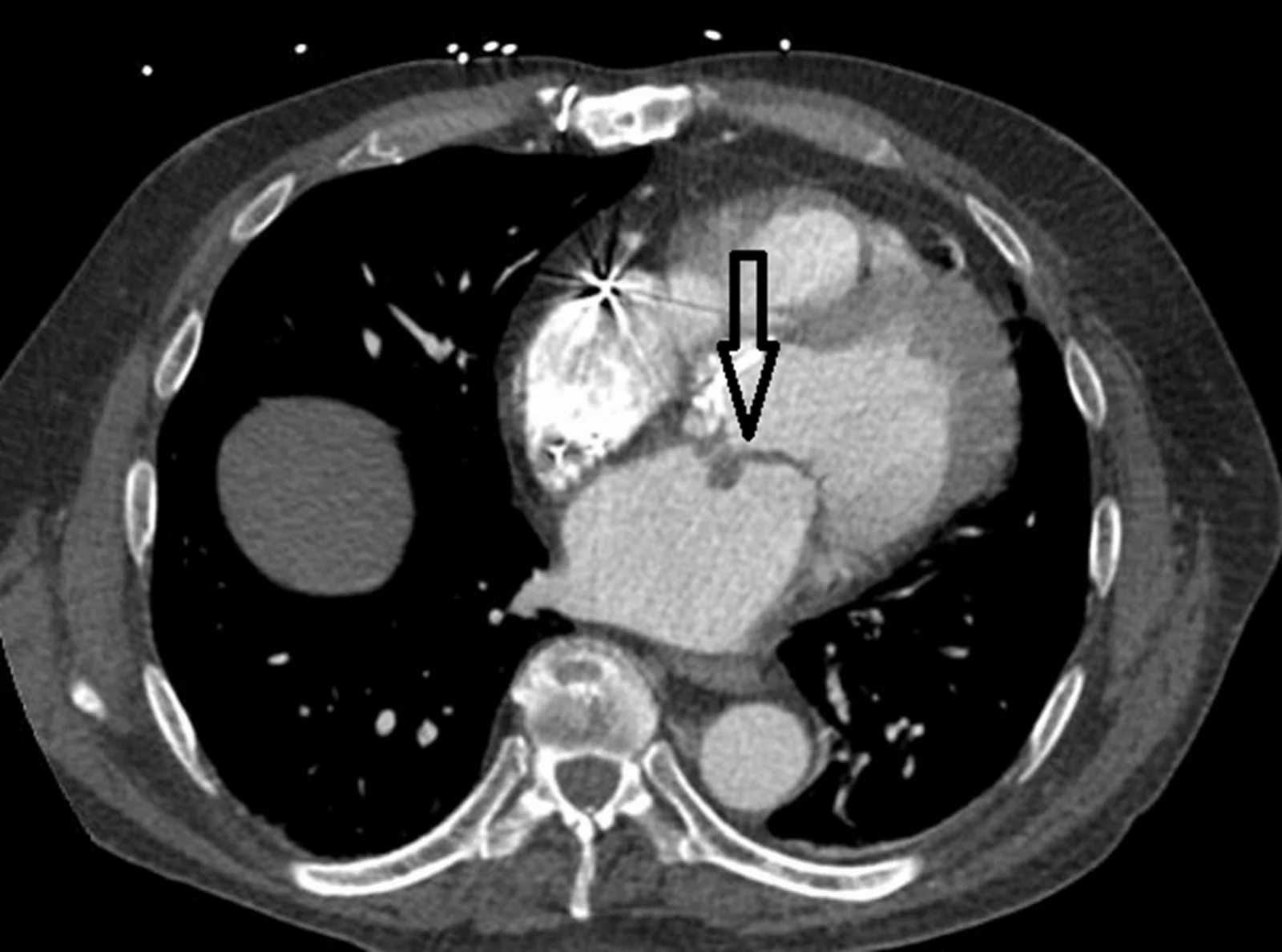
Wider axial CTA image The image shows a mitral valve abnormality in the context of cardiomegaly, left atrial enlargement, postoperative thoracic changes, and clearly visualized lung bases. The arrow highlights the mitral valve region requiring deliberate cardiac review on non-coronary CTA. CTA: Computed tomography angiography

The lungs were clear. Reactive-appearing mediastinal lymph nodes measured up to 1.1 cm in the right paratracheal station and 1.9 cm in the subcarinal station. A 6 mm filling defect along the left lateral wall of the distal trachea was also present on the prior CT examination and was considered possibly compatible with a tracheal polyp. No acute abdominal or pelvic vascular abnormality was identified. Incidental findings included bilateral renal cortical cysts, focal left renal cortical scarring, a duodenal diverticulum, fat-containing supraumbilical and umbilical hernias, and bilateral hip arthroplasties.

Clinical course

The patient was managed by the treating clinical teams in the setting of recurrent endocarditis, prior aortic graft infection, severe baseline comorbidity, and absence of CTA evidence of aorto-bronchial fistula. According to the most recent available clinical note, the patient was being discharged. Detailed blood culture results, inflammatory markers, antimicrobial regimen, and a contemporaneous surgical or pathology confirmation of the mitral valve finding were not available for inclusion in this report.

## Discussion

This case is relevant for both radiologists and clinicians because the CTA was requested for an urgent non-valvular indication: hemoptysis with concern for aorto-bronchial fistula in a patient with prior TEVAR and suspected graft infection. The primary vascular question was answered negatively, but the examination also demonstrated a destructive mitral valve abnormality. In high-risk IE patients, a negative CTA for the primary suspected complication should not end image review; cardiac valves, prosthetic material, peri-graft spaces, and extracardiac embolic or infectious complications should be assessed systematically.

The most distinctive feature of the case is that anterior mitral leaflet perforation was detected after several echocardiographic examinations during the same year. This observation should be interpreted carefully. Echocardiography remains the first-line and most sensitive method for many valvular manifestations of IE. Prior reports may fail to describe a lesion because of acoustic window limitations, prosthetic material, device artifact, complex postoperative anatomy, lesion evolution after the examination, or interpretive focus on other clinically dominant findings. 

Published comparative data support this balanced interpretation. In a systematic review and meta-analysis comparing CT and TEE in IE, TEE showed higher sensitivity than CT for vegetation detection and leaflet perforation, whereas CT contributed important information in selected anatomic and prosthetic contexts [3]. A contemporary comparative meta-analysis similarly reported that TEE was more sensitive for leaflet perforation, while cardiac CT had value in the evaluation of valvular and perivalvular complications, particularly as part of multimodality assessment [4]. These data support using CT as an adjunct rather than a replacement for echocardiography.

The updated 2023 Duke-International Society for Cardiovascular Infectious Diseases criteria recognize the expanding role of imaging in IE diagnosis. The updated major imaging criterion includes echocardiography or cardiac CT findings such as vegetation, valvular or leaflet perforation, aneurysm, abscess, pseudoaneurysm, intracardiac fistula, and significant new valvular regurgitation [1]. The 2023 European Society of Cardiology guidelines also emphasize multimodality imaging, including echocardiography, CT, nuclear imaging, and other modalities depending on the suspected complication and type of implanted material [2]. In the present case, CTA demonstrated two morphologic features that are meaningful in this framework: a focal anterior mitral leaflet defect and an adjacent low-attenuation lesion suspicious for vegetation.

The CT appearance deserves specific comment. Vegetation is usually seen on CT as a low-attenuation mobile or sessile mass attached to a valve or endocardial surface, although motion artifact and lack of electrocardiographic gating may limit evaluation. Leaflet perforation may appear as a focal discontinuity, defect, or contrast passage through a valve leaflet, and correlation with echocardiographic color Doppler is often necessary to confirm the hemodynamic consequence. In this patient, the abnormality was identified on a thoracoabdominal CTA rather than a dedicated electrocardiographically gated cardiac CT. Despite this limitation, the finding was conspicuous enough to warrant reporting because of the patient's recurrent IE, severe mitral regurgitation, and adjacent suspected vegetation.

The clinical context also increases the importance of the finding. Recurrent IE in a patient with prior prosthetic valve endocarditis, infected aortic graft or TEVAR history, chronic osteomyelitis, chronic prosthetic joint infection, and intracardiac device leads creates multiple potential reservoirs or substrates for persistent infection. In such patients, destructive lesions may involve valves beyond the initially infected prosthetic aortic valve. Recognition of mitral involvement may prompt further clinical and imaging assessment, including multidisciplinary evaluation.

This case has limitations. The 2022 surgical pathology report was not available. Detailed microbiologic data, inflammatory markers, antimicrobial regimen, and direct surgical or pathologic confirmation of the mitral valve finding were not available. The prior echocardiographic reports did not describe mitral perforation, but the original TEE and TTE images were not available for retrospective comparison. Finally, the CTA was not a dedicated electrocardiographically gated cardiac CT study, which may reduce confidence for small valve lesions. Nevertheless, the combination of a focal anterior mitral leaflet defect, adjacent hypoattenuating lesion, recurrent IE, and severe mitral regurgitation makes the finding clinically important and appropriate to highlight.

## Conclusions

Thoracoabdominal CTA performed for hemoptysis and suspected aorto-bronchial fistula can reveal clinically important valvular complications in patients with recurrent IE and complex prosthetic cardiovascular material. In this patient, nongated thoracoabdominal CTA showed no aorto-bronchial fistula but demonstrated a focal anterior mitral leaflet defect with an adjacent hypoattenuating lesion, raising concern for leaflet perforation and vegetation, a finding not reported on multiple echocardiographic examinations performed earlier in the same year. Confirmatory surgical, pathological, and targeted echocardiographic data were unavailable.

The case reinforces the complementary role of CT in IE. Echocardiography remains central for valvular assessment, but careful review of the mitral and aortic valves on CTA is essential when patients have recurrent infection, prior prosthetic valve endocarditis, aortic graft infection, and implanted cardiac hardware. A structured cardiac search pattern on non-coronary CTA may help identify destructive valve complications requiring further clinical and imaging assessment.
